# Aspiration and Injection of Morphin-Eucain in Tetanus

**Published:** 1904-10

**Authors:** 


### Aspiration and Injection of Morphin-Eucain in Tetanus.

John B. Murphy, Chicago, (*Journal of the American Medical Asso.*, Aug. 13, 1904,) says: a patient treated eight days after infection was given three full doses of antitetanic serum, but without effect; the convulsions increased and were almost continuous. Thereupon a lumbar puncture was made and 16 c.c. of cerebrospinal fluid withdrawn. At the same time 3 c.c. of the following was injected:

| | |
|---|---|
| Beta-eucain | 1½ grains. |
| Morphin sulphate | ⅓ " |
| Sodium chloride | 3 " |
| Distilled water | 3½ " |

This had been sterilised by boiling. The patient slept 4 hours immediately and through the night 1½ hours at a time. There were only 8 spasms in the succeeding 24 hours. A more severe spasm recurring the next morning, another puncture was made, 15 c.c. of fluid withdrawn, and 4 c.c. of the above injected. This was repeated on the two following days and then, with intervals of two days, two more aspirations and injections were made. He was discharged as cured 10 days later.

The quantities of morphine and eucain used were exceedingly small as this was the writer's first case. There was no sweating, headache or collapse, symptoms frequently noticed after lumbar injection of cocain. He believes the eucain should be increased to ⅙ to ⅓ grain at each injection, and this treatment might be made more frequent. Eucain is much safer than cocain, as it admits of boiling and there is less idiosyncrasy to intoxication. Reduction of the spasms prevents death from exhaustion or interference with respiration. The diminution of pus in the aspired fluid would lead one to believe that lessening of pressure aided the fluid in overcoming infection. There is no reason why the cerebrospinal cavities cannot be washed out by salt or other neutralising solutions.

